# Supplementary material for: Semi-Synthesis and In Vitro Anti-Cancer Evaluation of Magnolol Derivatives
Source: Molecules. 2021 Jul 15;26(14):4302. doi: 10.3390/molecules26144302 (PMC8303457; doi:10.3390/molecules26144302)
Supplement: Supplementary file 1 [file molecules-26-04302-s001.zip › molecules-1284542-SI.pdf]

Supplementary materials for

Semi-Synthesis and In Vitro Anti-Cancer Evaluation of Magnolol Derivatives

Xiao-Long Sun [1], Mei-Lin Zhu [1], Yi-Qun Dai [1], Hong-Mei Li [1], Bo-Han Li [1], Hui Ma [1], Chang-Hao Zhang [2,*] and Cheng-Zhu Wu [1,*]

[1] School of Pharmacy, Bengbu Medical College, 2600 Donghai Road, Bengbu 233030, China;
sunxiaolong96@foxmail.com (X.-L.S.); zlyk521@126.com (M.-L.Z.);
daiyiqun25@126.com (Y.-Q.D.);
athongmei@bbmc.edu.cn (H.-M.L.); libohan1228@foxmail.com (B.-H.L.);
mahui9513@foxmail.com (H.M.)

[2] Key Laboratory of Natural Medicines of the Changbai Mountain, Ministry of Education, College of Pharmacy, Yanbian University, 977 Gongyuan Road, Yanji 133002, China

* Correspondence: zhangch@ybu.edu.cn (C.-H.Z.);
wuchengzhu0611@bbmc.edu.cn (C.-Z.W.);
Tel.: +86-443-243-6006(C.-H.Z.); +86-552-317-5232(C.-Z.W.)

Figure S1 ^{1}H NMR spectrum of compound 1a 3
Figure S2 ^{13}C NMR spectrum of 1a 3
Figure S3 ^{1}H NMR spectrum of compound 1b 4
Figure S4 ^{13}C NMR spectrum of 1b 4
Figure S5 ^{1}H NMR spectrum of compound 2a 5
Figure S6 ^{13}C NMR spectrum of 2a 5
Figure S7 ^{1}H NMR spectrum of compound 2b 6
Figure S8 ^{13}C NMR spectrum of 2b 6
Figure S9 ^{1}H NMR spectrum of compound 3a 7
Figure S10 ^{13}C NMR spectrum of 3a 7
Figure S11 ^{1}H NMR spectrum of compound 3b 8
Figure S12 ^{13}C NMR spectrum of 3b 8
Figure S13 ^{1}H NMR spectrum of compound 4a 9
Figure S14 ^{13}C NMR spectrum of 4a 9
Figure S15 ^{1}H NMR spectrum of compound 4b 10
Figure S16 ^{13}C NMR spectrum of 4b 10
Figure S17 ^{1}H NMR spectrum of compound 5a 11
Figure S18 ^{13}C NMR spectrum of 5a 11
Figure S19 ^{1}H NMR spectrum of compound 5b 12
Figure S20 ^{13}C NMR spectrum of 5b 12
Figure S21 ^{1}H NMR spectrum of compound 6a 13
Figure S22 ^{13}C NMR spectrum of 6a 13
Figure S23 ^{1}H NMR spectrum of compound 6b 14
Figure S24 ^{13}C NMR spectrum of 6b 14
Figure S25 ^{1}H NMR spectrum of compound 7a 15
Figure S26 ^{13}C NMR spectrum of 7a 15
Figure S27 ^{1}H NMR spectrum of compound 8a 16
Figure S28 ^{13}C NMR spectrum of 8a 16
Figure S29 ^{1}H NMR spectrum of compound 9a 17
Figure S30 ^{13}C NMR spectrum of 9a 17
Figure S31 HR-ESI-MS spectrum of compound 3a 18
Figure S32 HR-ESI-MS spectrum of compound 3b 18
Figure S33 HR-ESI-MS spectrum of compound 4a 19
Figure S34 HR-ESI-MS spectrum of compound 4b 19
Figure S35 HR-ESI-MS spectrum of compound 5a 20
Figure S36 HR-ESI-MS spectrum of compound 5b 20
Figure S37 HR-ESI-MS spectrum of compound 6a 21
Figure S38 HR-ESI-MS spectrum of compound 6b 21
Figure S39 HR-ESI-MS spectrum of compound 7a 22
Figure S40 HR-ESI-MS spectrum of compound 8a 22
Figure S41 HR-ESI-MS spectrum of compound 9a 23

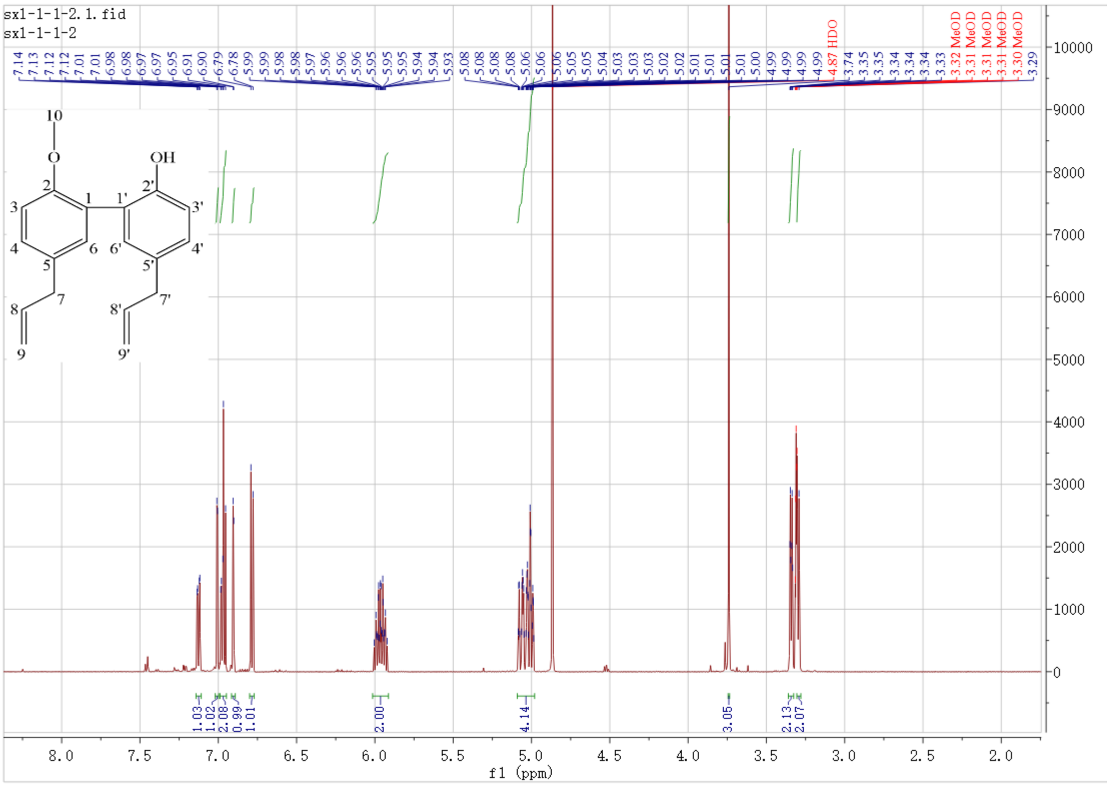

Figure S1 ^{1}H NMR spectrum of compound 1a

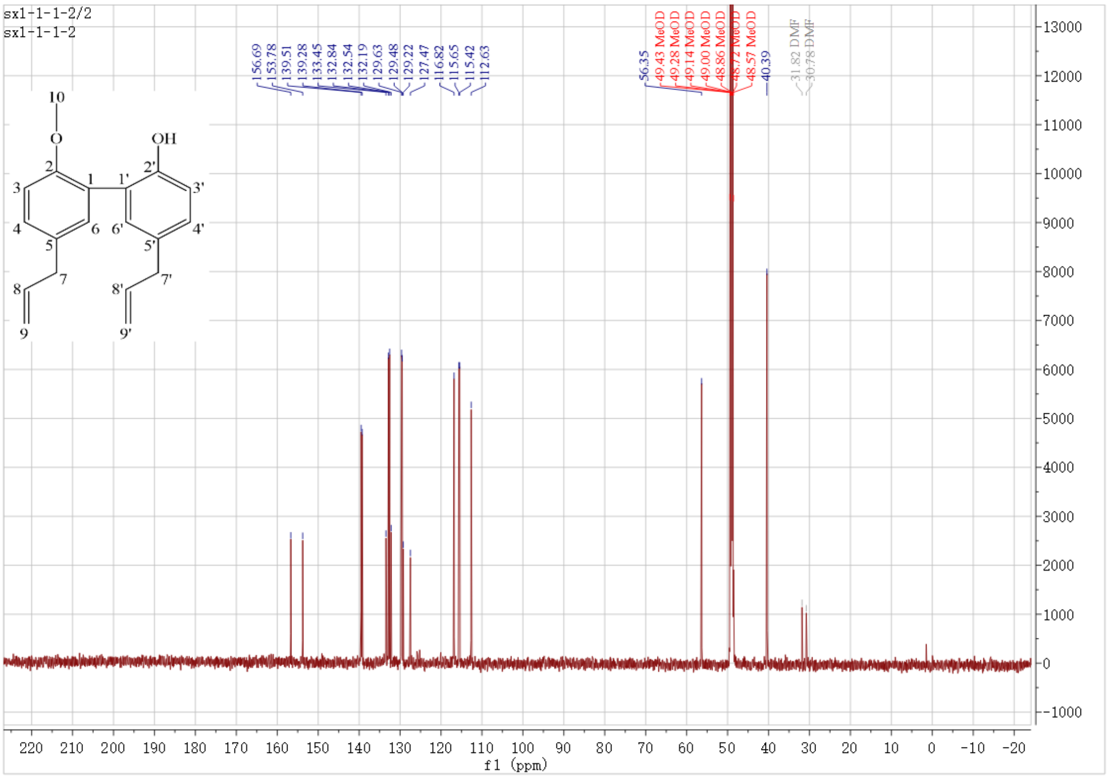

Figure S2 ^{13}C NMR spectrum of 1a

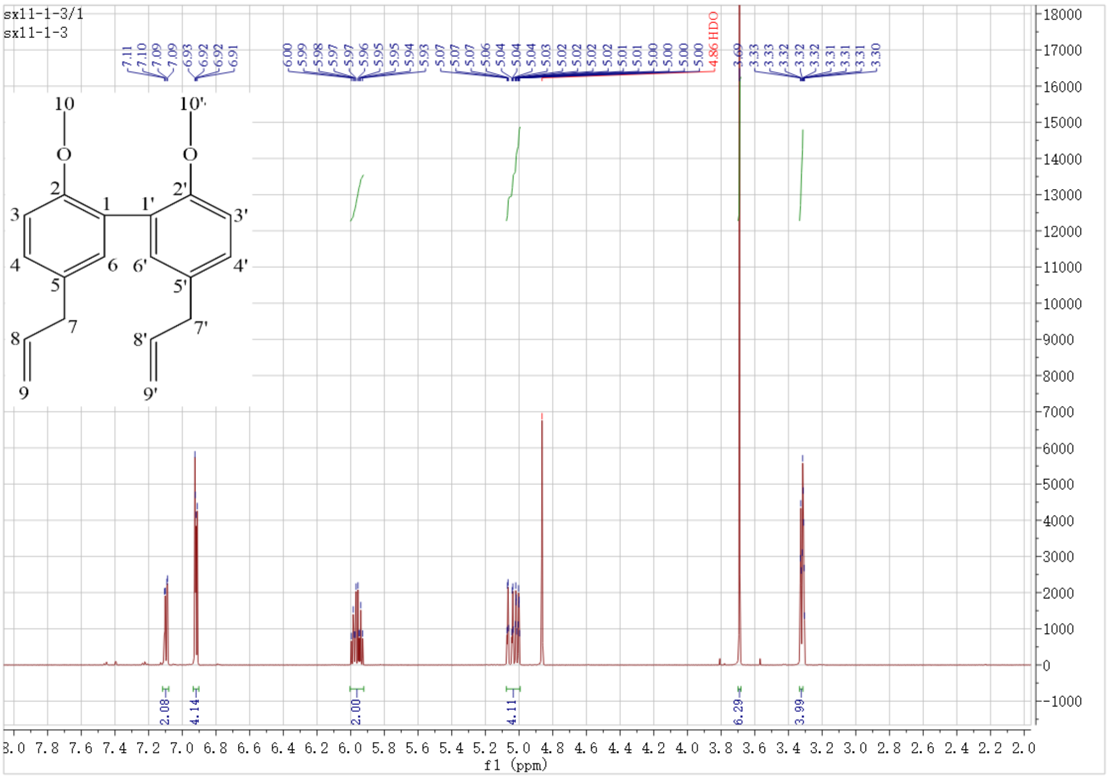

Figure S3 ^{1}H NMR spectrum of compound 1b

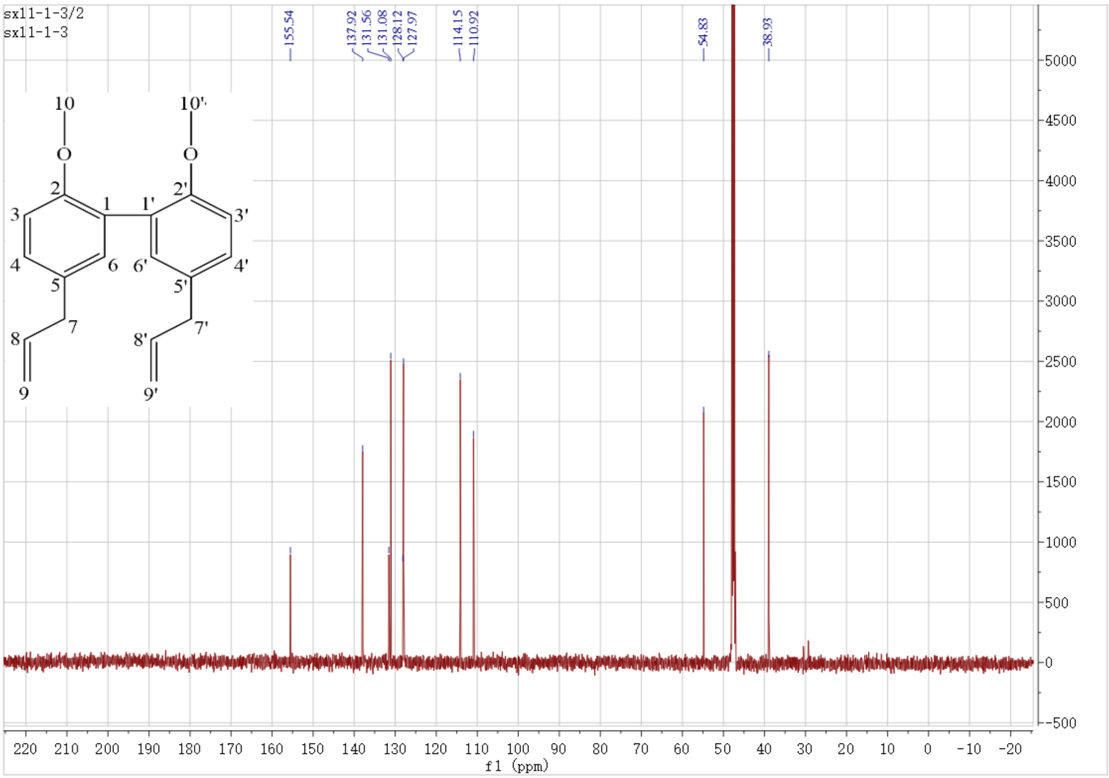

Figure S4 ^{13}C NMR spectrum of 1b

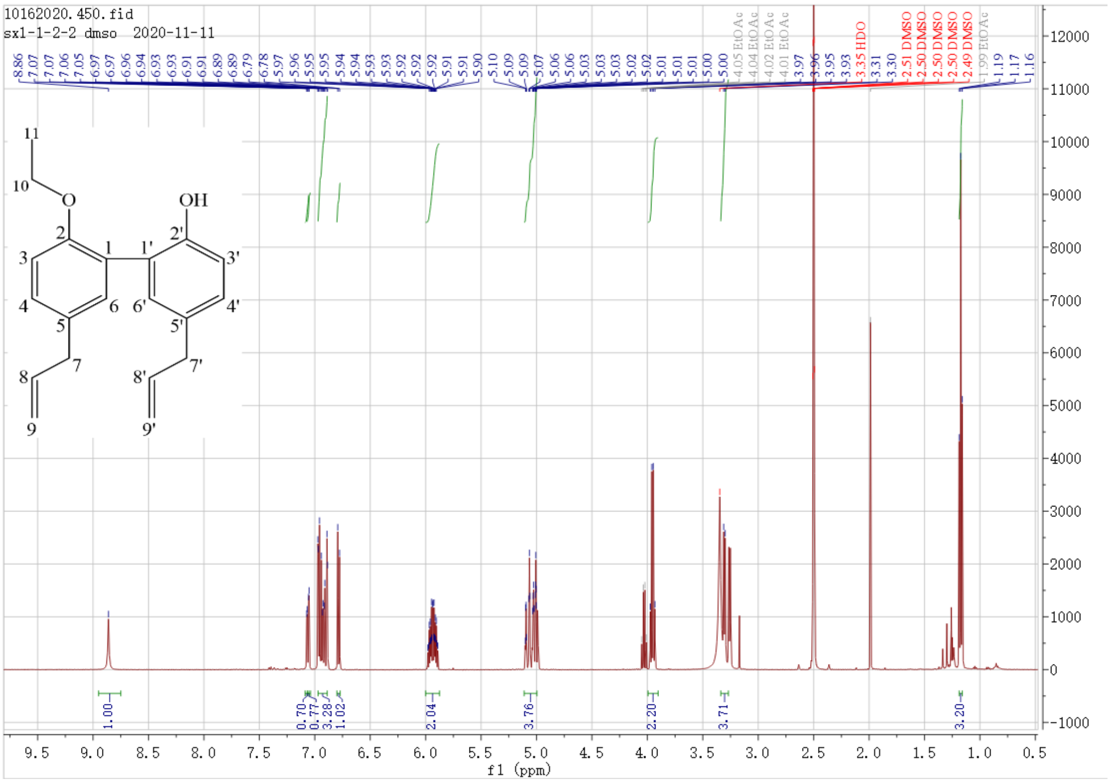

Figure S5 ^{1}H NMR spectrum of compound 2a

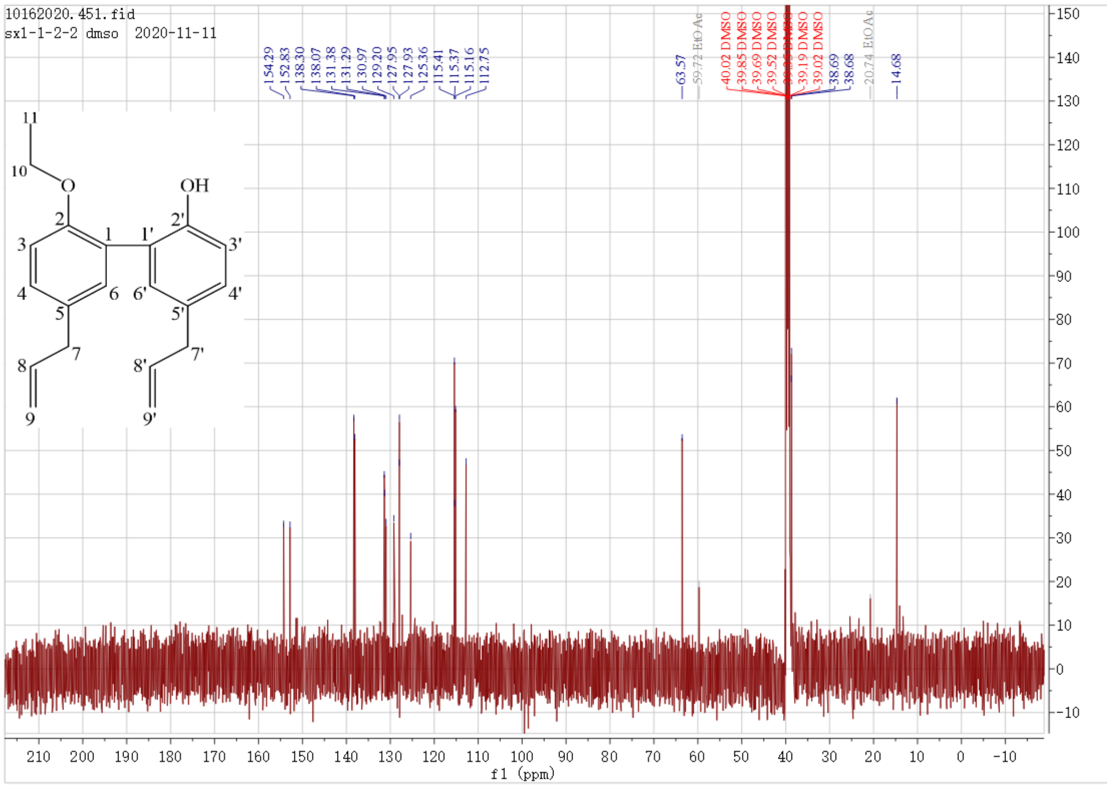

Figure S6 ^{13}C NMR spectrum of 2a

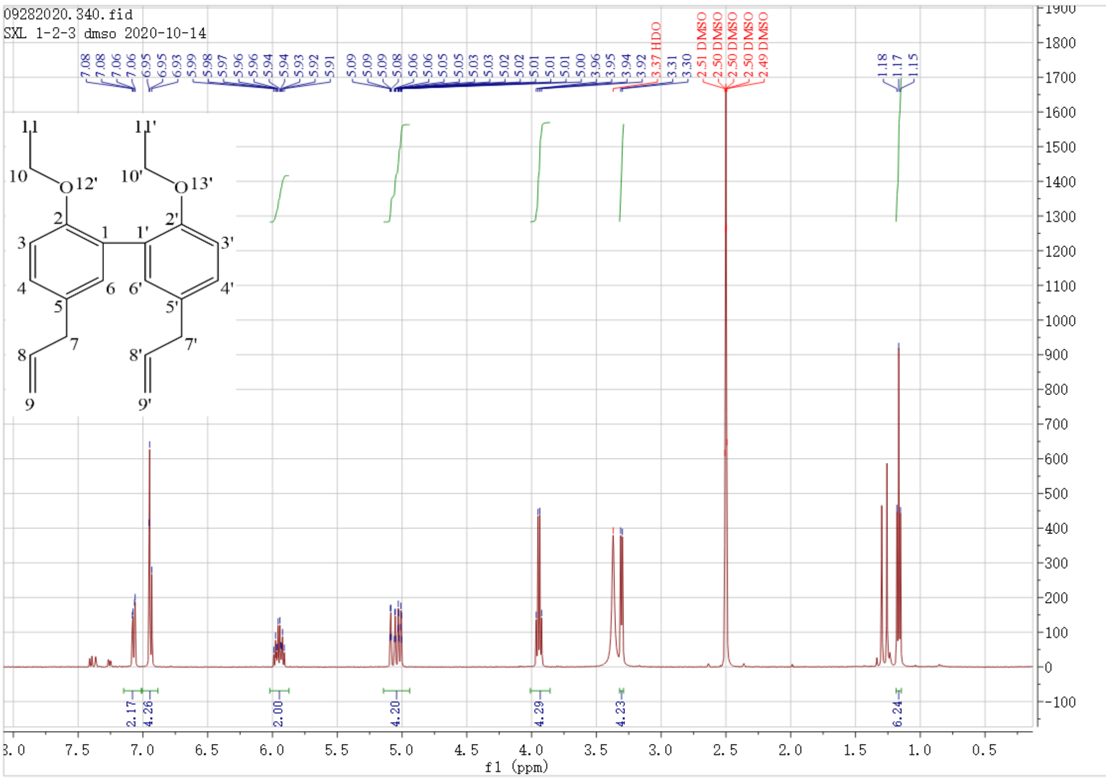

Figure S7 ^{1}H NMR spectrum of compound 2b

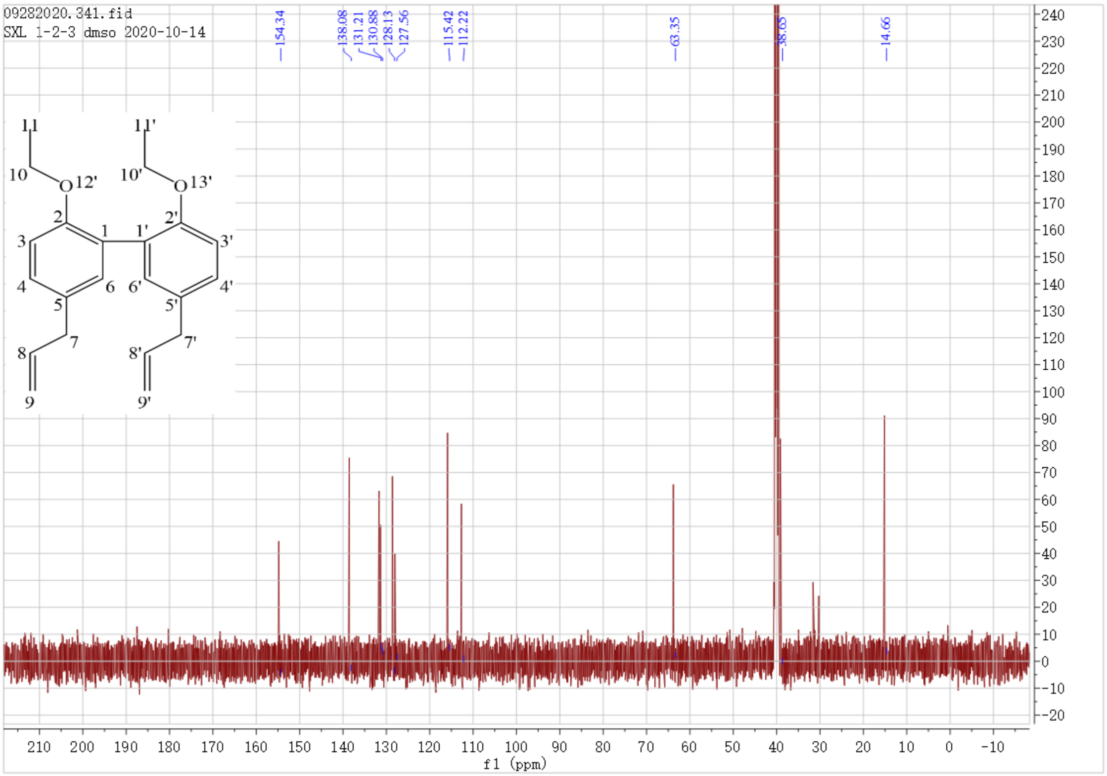

Figure S8 ^{13}C NMR spectrum of 2b

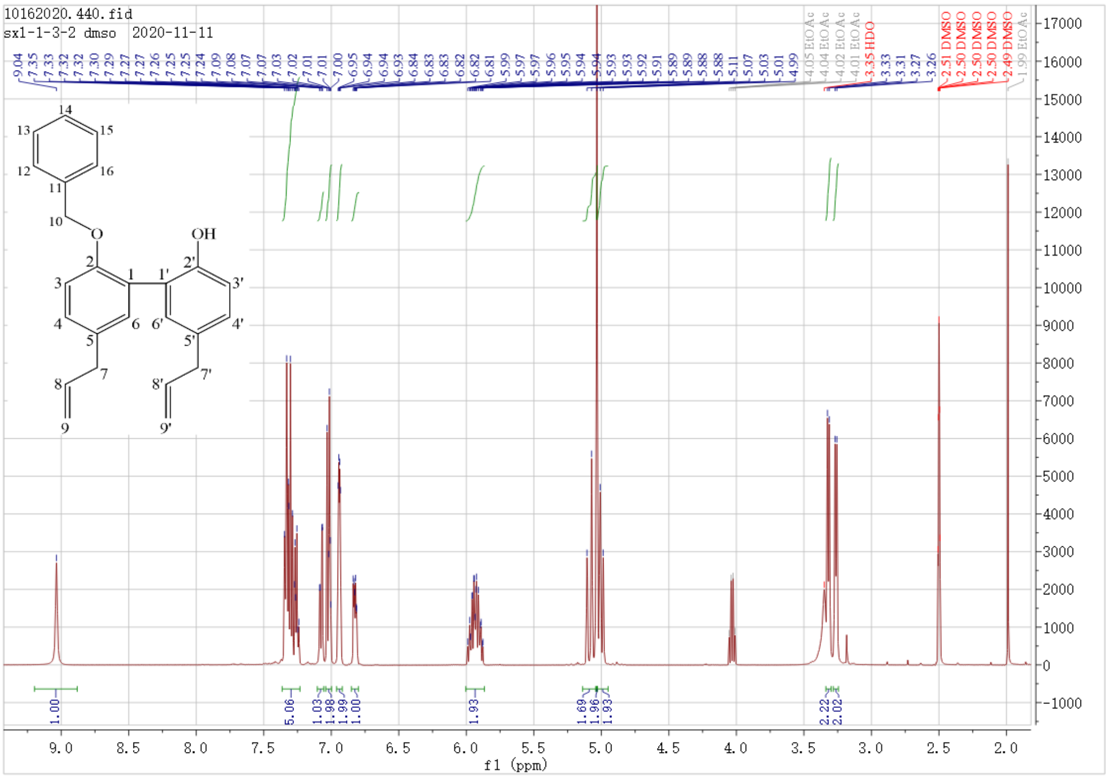

Figure S9 ^{1}H NMR spectrum of compound 3a

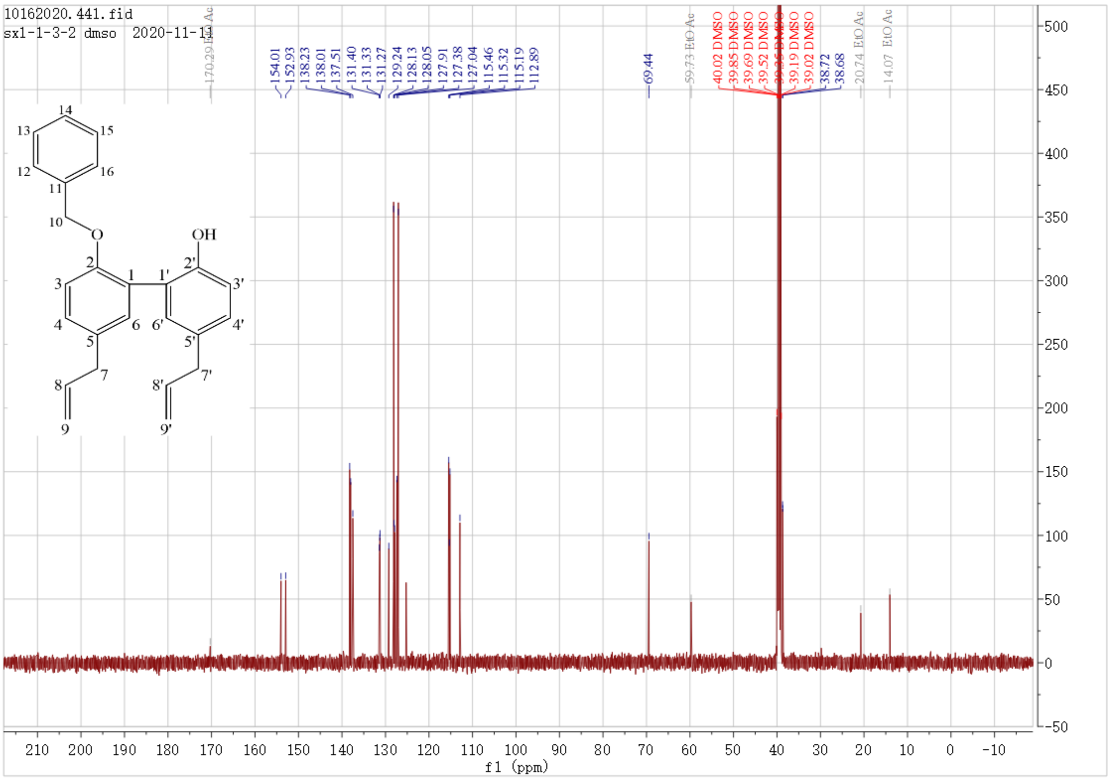

Figure S10 ^{13}C NMR spectrum of 3a

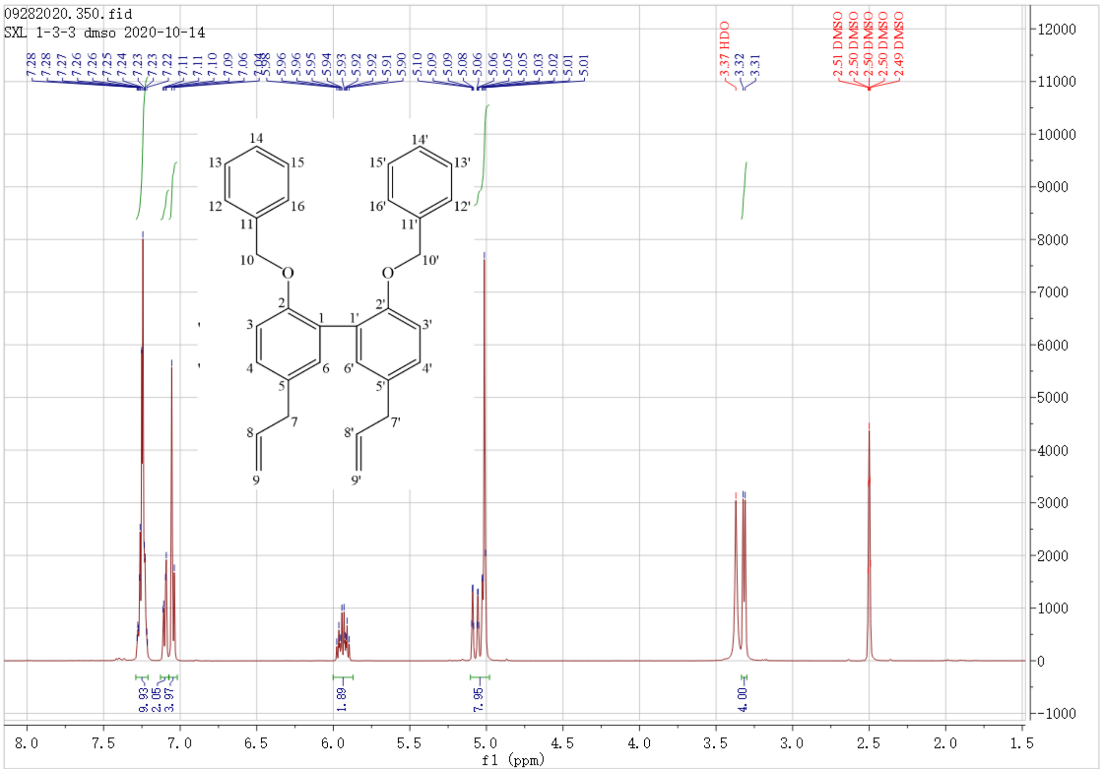

Figure S11 ^{1}H NMR spectrum of compound 3b

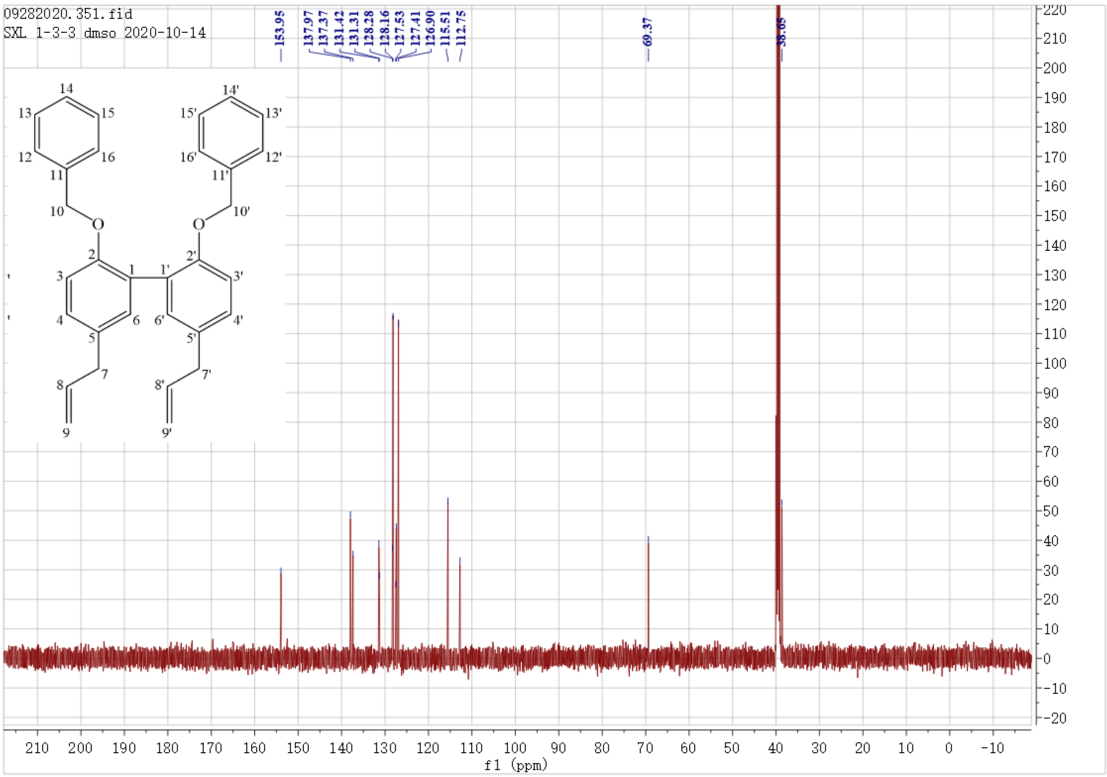

Figure S12 ^{13}C NMR spectrum of 3b

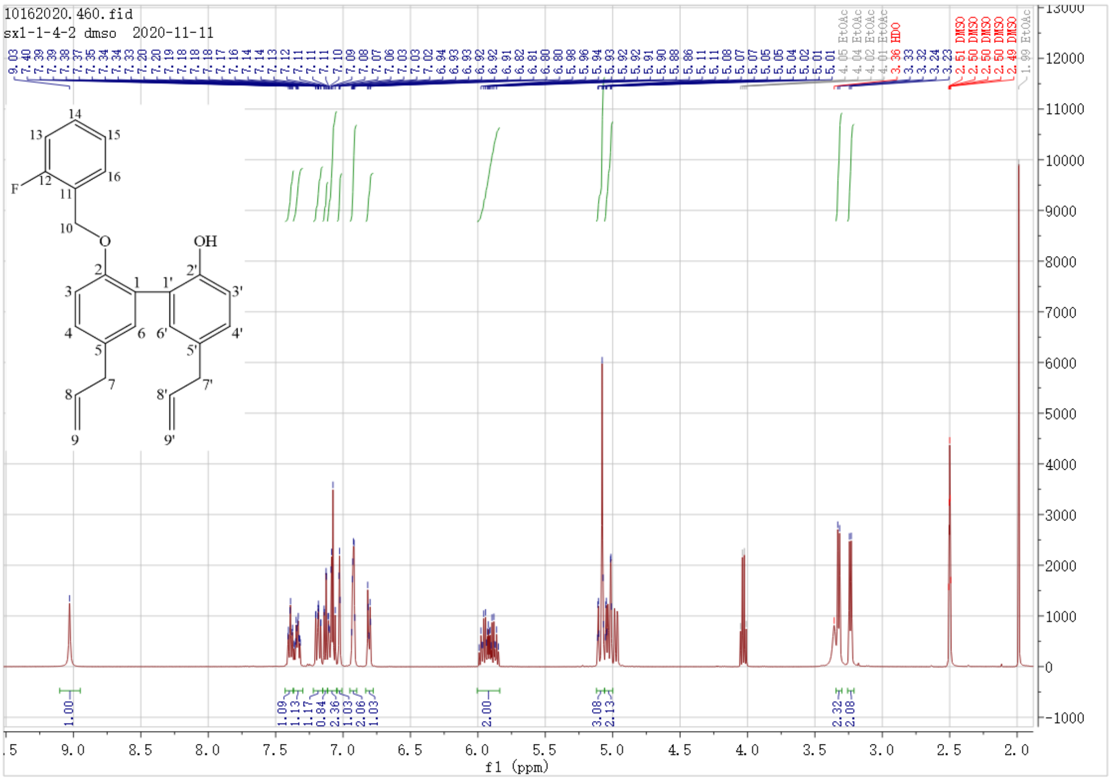

Figure S13 ^{1}H NMR spectrum of compound 4a

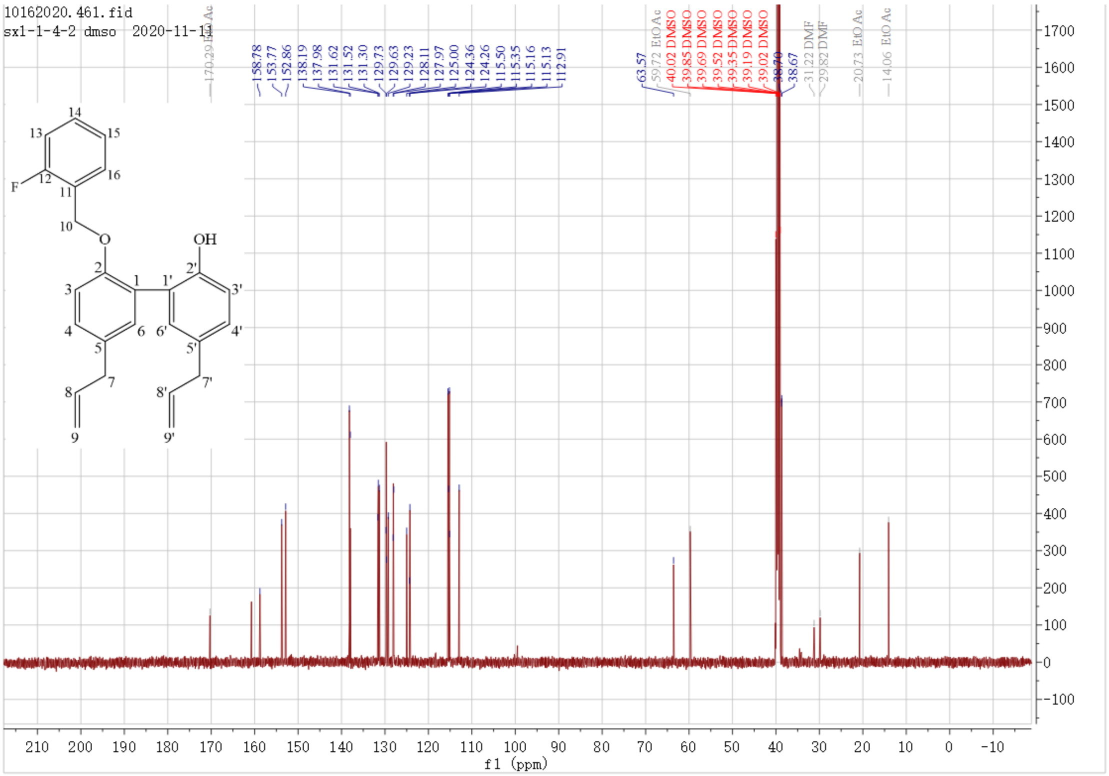

Figure S14 ^{13}C NMR spectrum of 4a

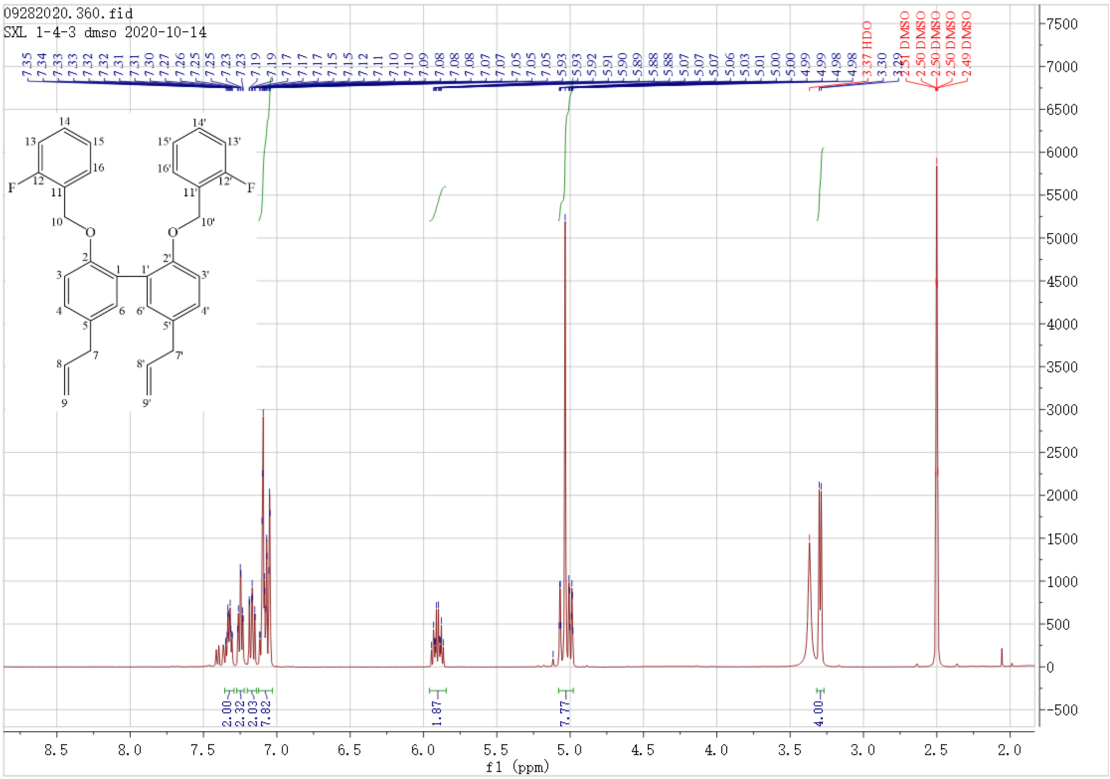

Figure S15 ^{1}H NMR spectrum of compound 4b

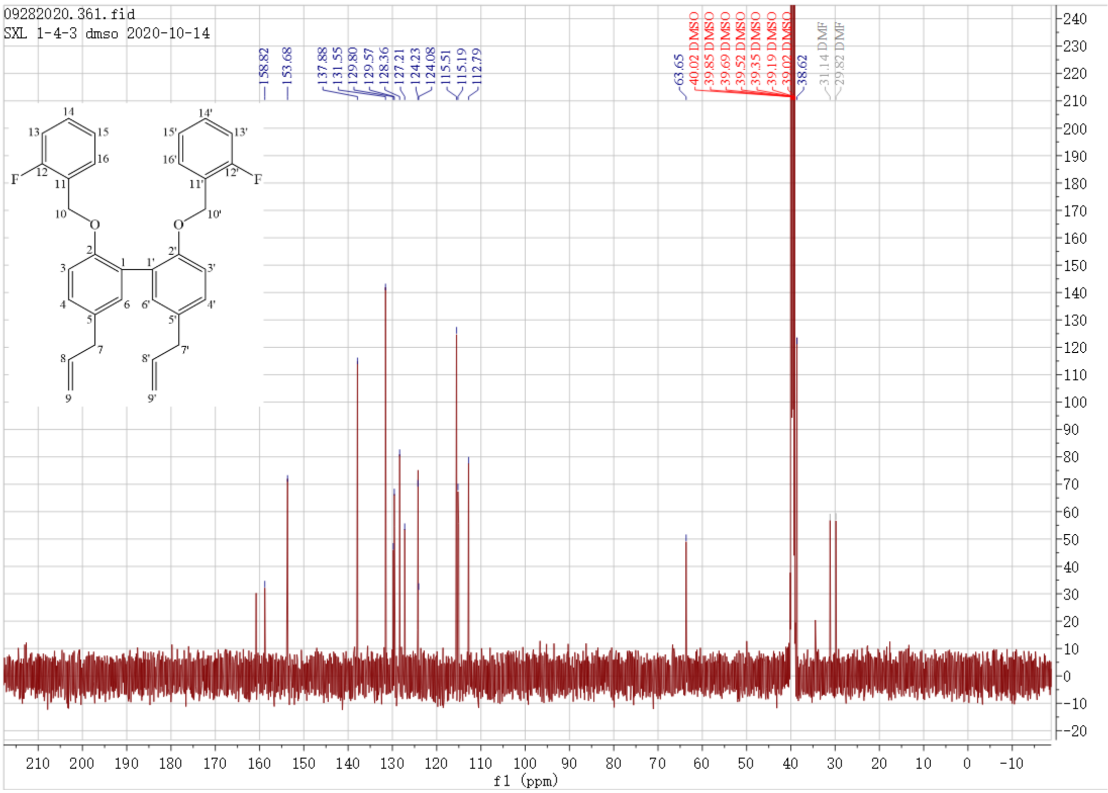

Figure S16 ^{13}C NMR spectrum of 4b

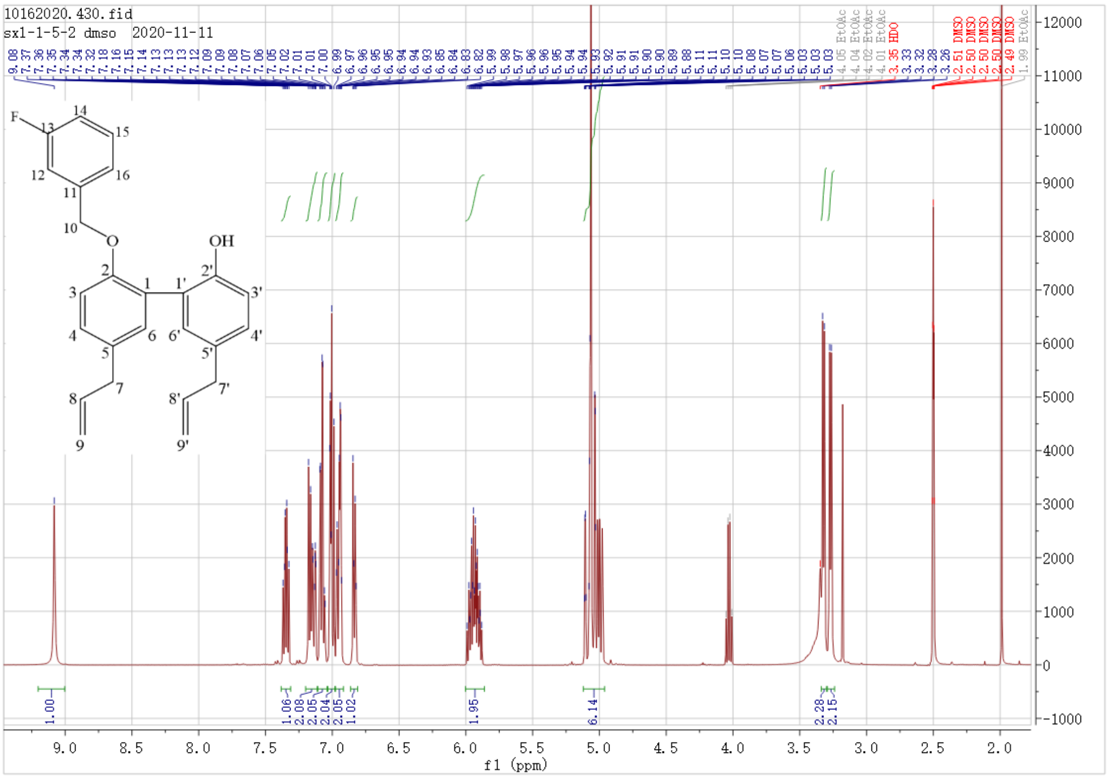

Figure S17 ^{1}H NMR spectrum of compound 5a

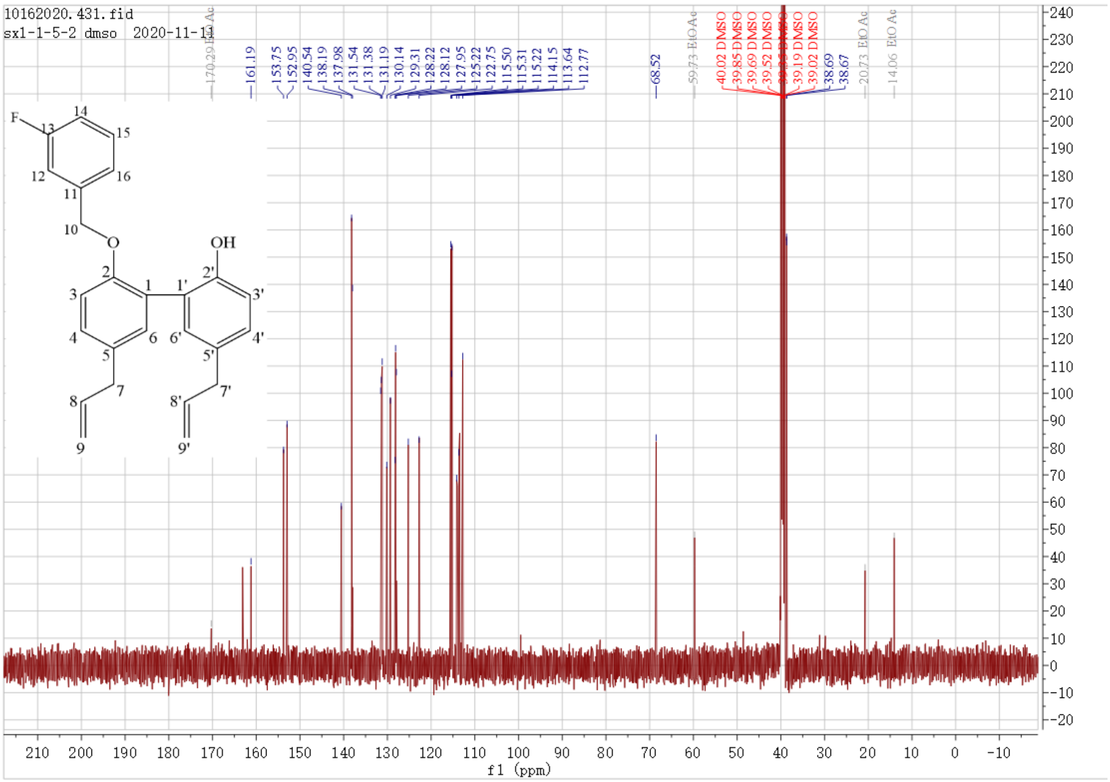

Figure S18 ^{13}C NMR spectrum of 5a

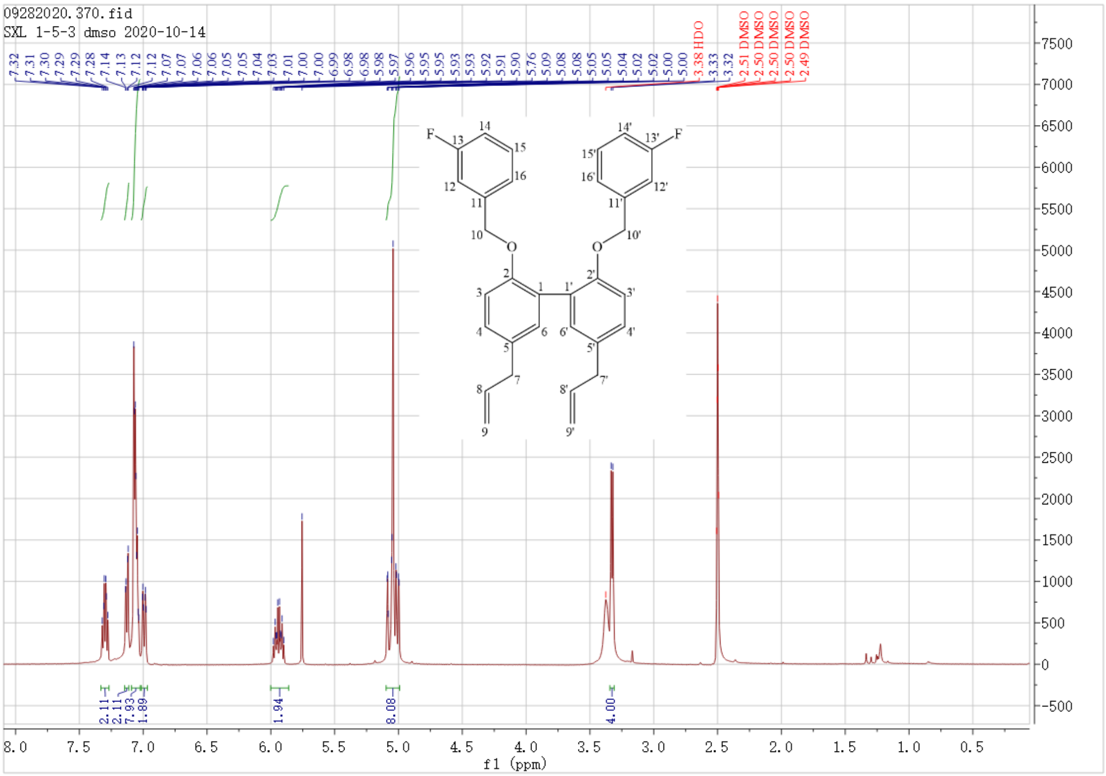

Figure S19 ^{1}H NMR spectrum of compound 5b

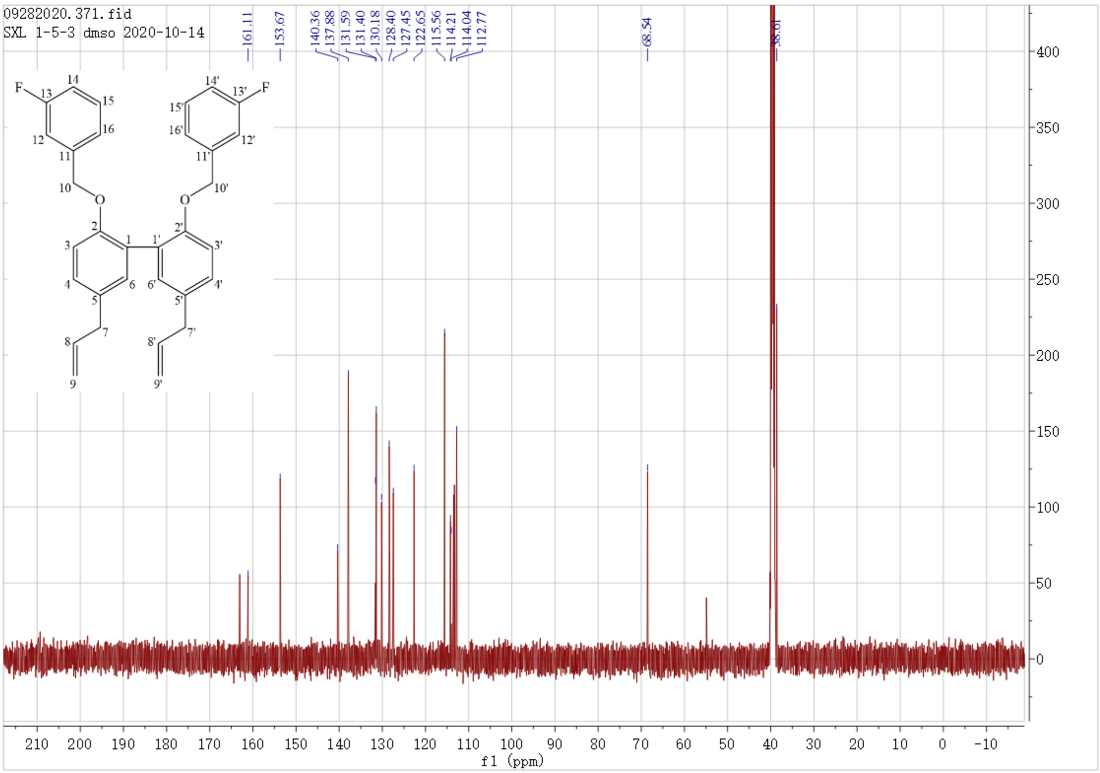

Figure S20 ^{13}C NMR spectrum of 5b

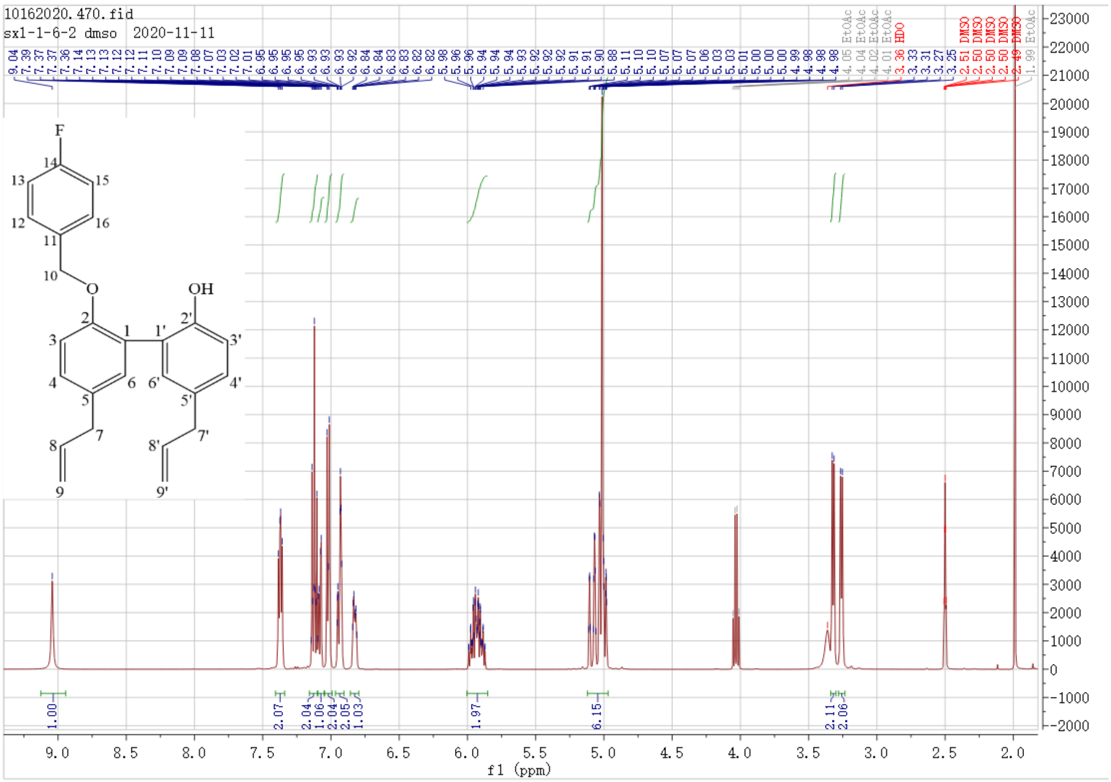

Figure S21 ^{1}H NMR spectrum of compound 6a

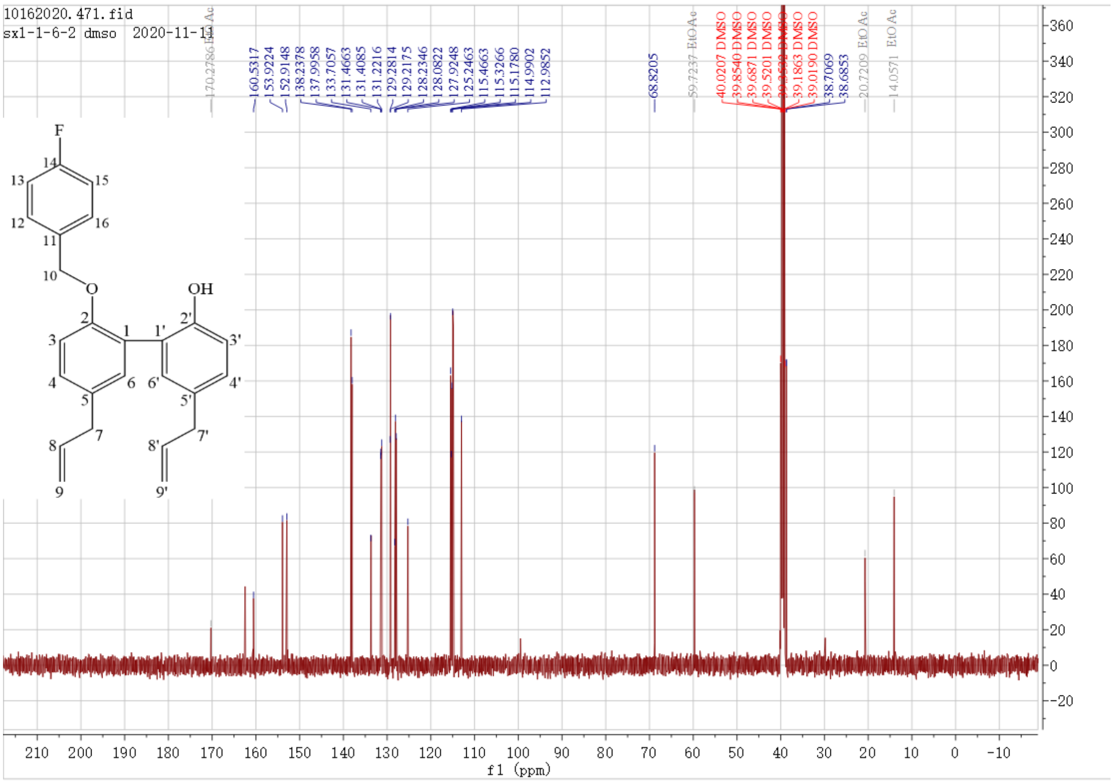

Figure S22 ^{13}C NMR spectrum of 6a

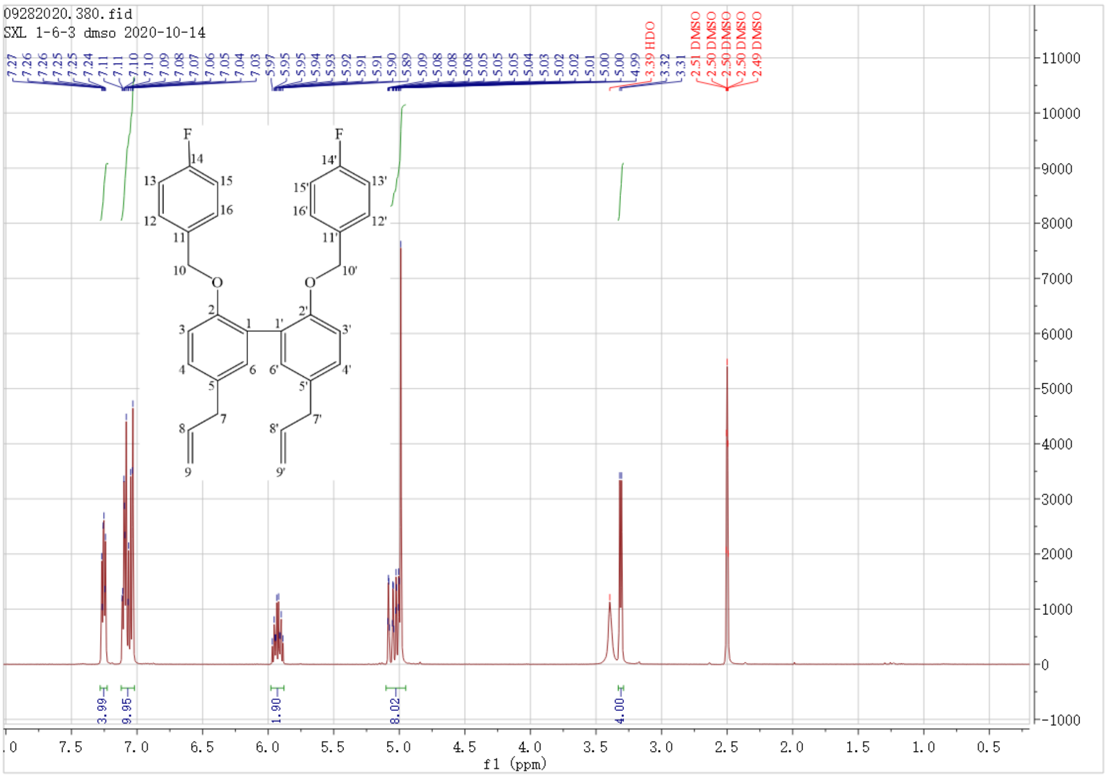

Figure S23 ^{1}H NMR spectrum of compound 6b

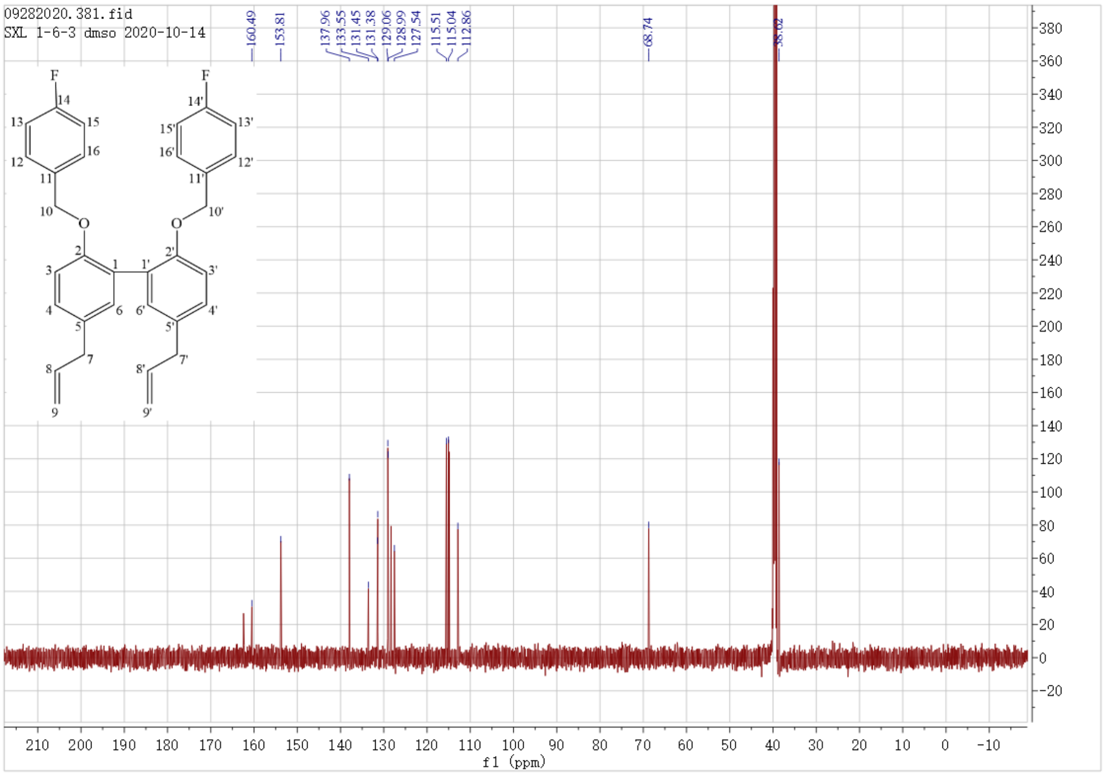

Figure S24 ^{13}C NMR spectrum of 6b

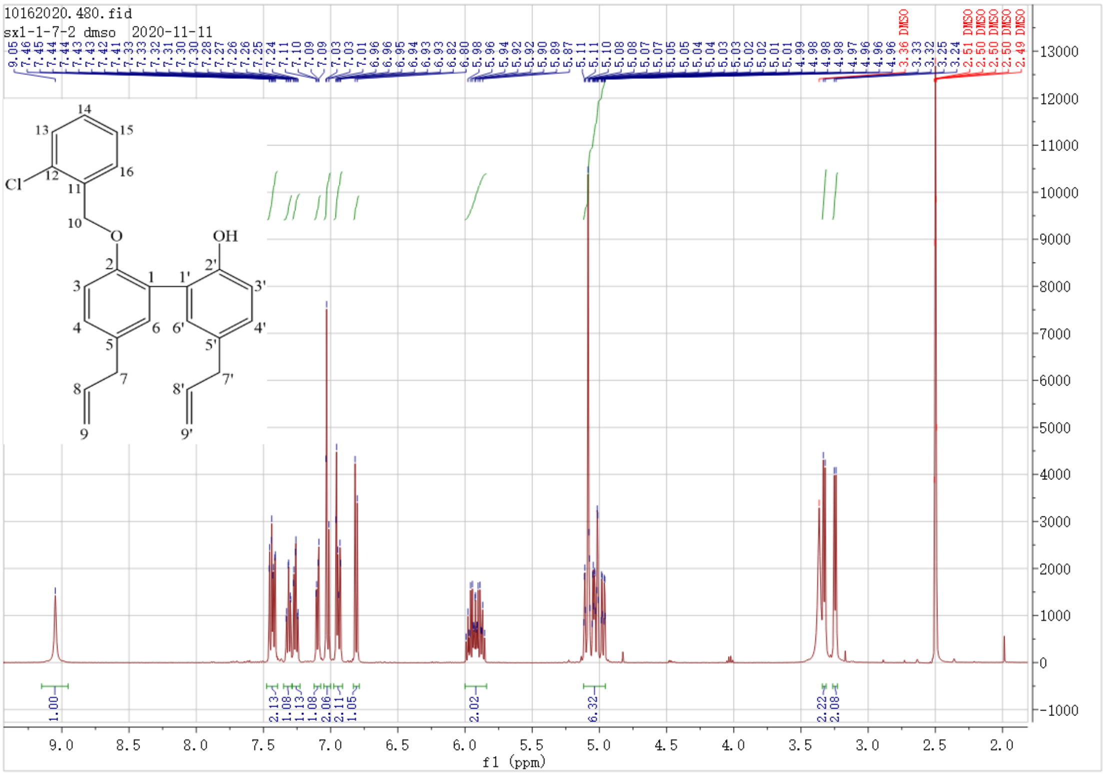

Figure S25 ^{1}H NMR spectrum of compound 7a

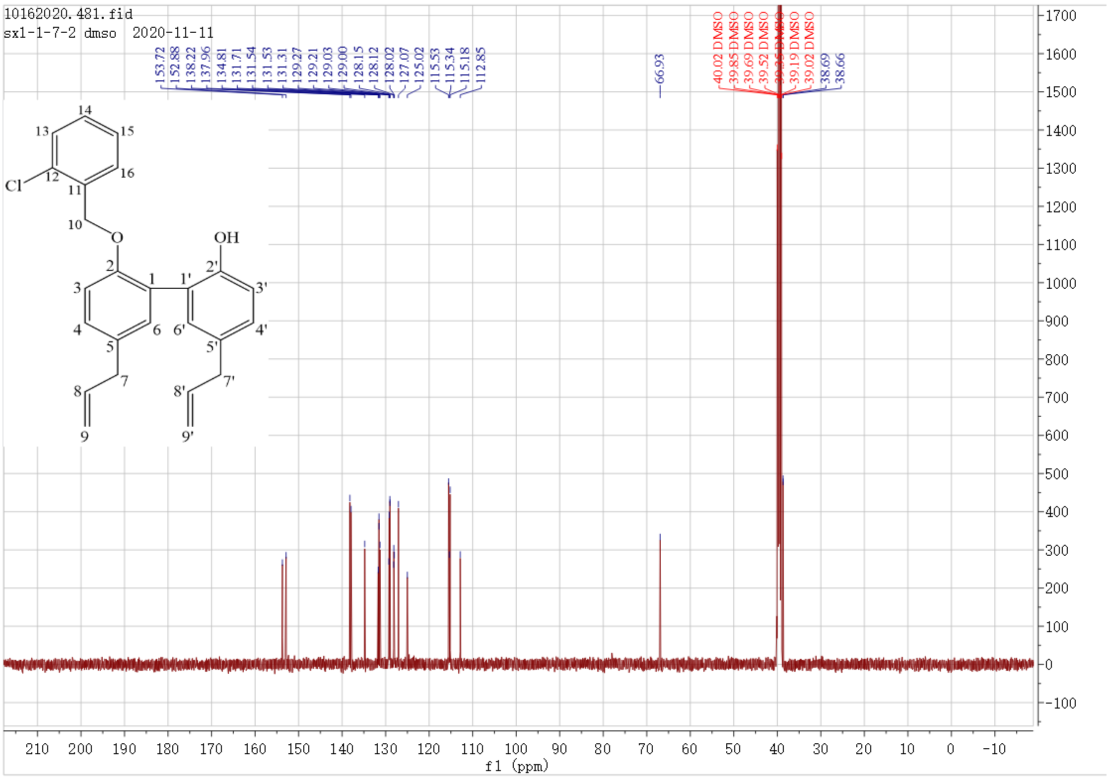

Figure S26 ^{13}C NMR spectrum of 7a

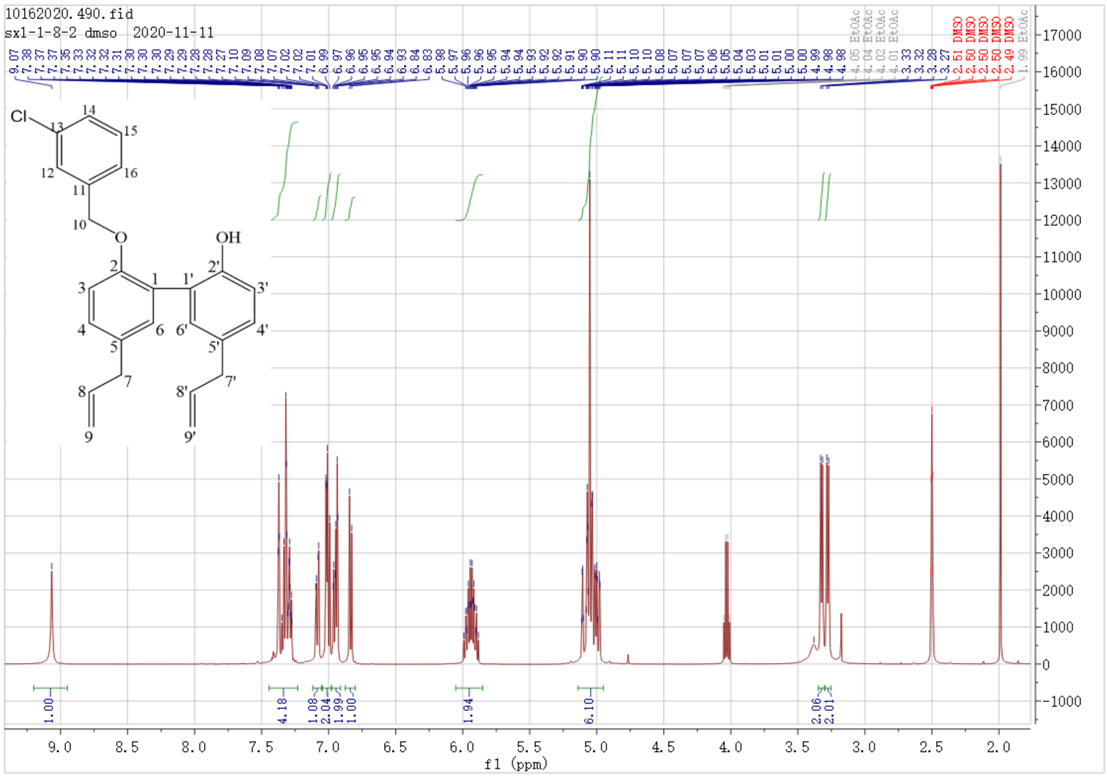

Figure S27 ^{1}H NMR spectrum of compound 8a

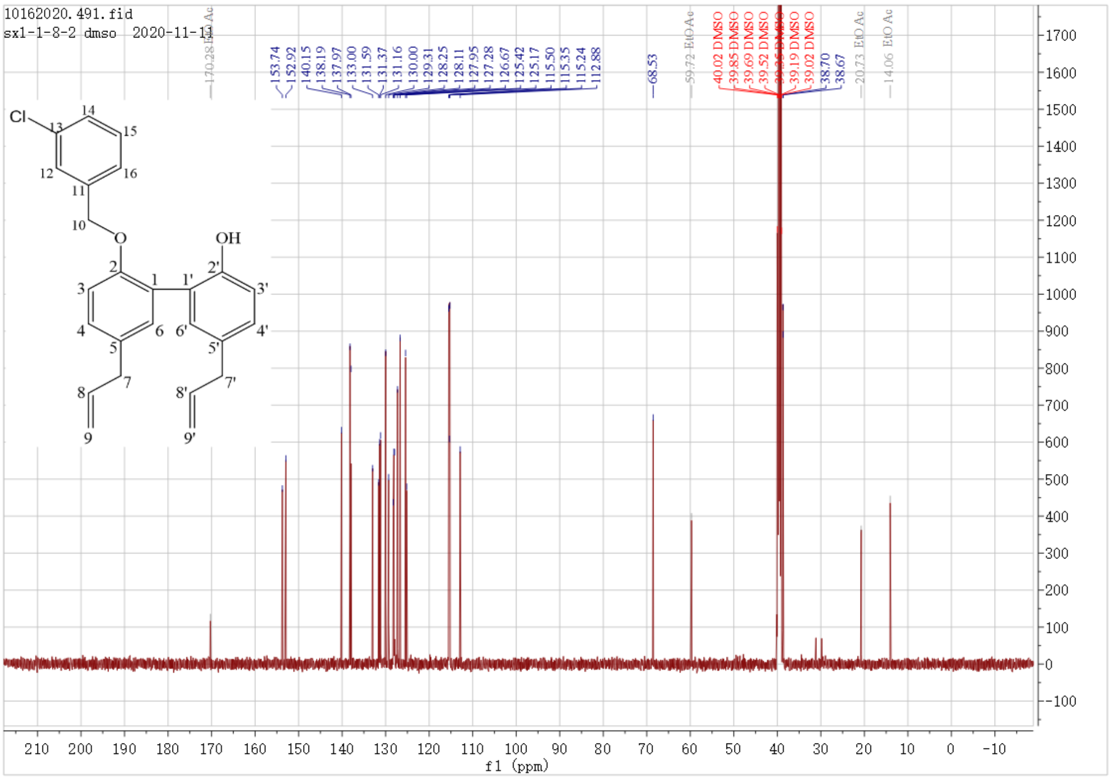

Figure S28 ^{13}C NMR spectrum of 8a

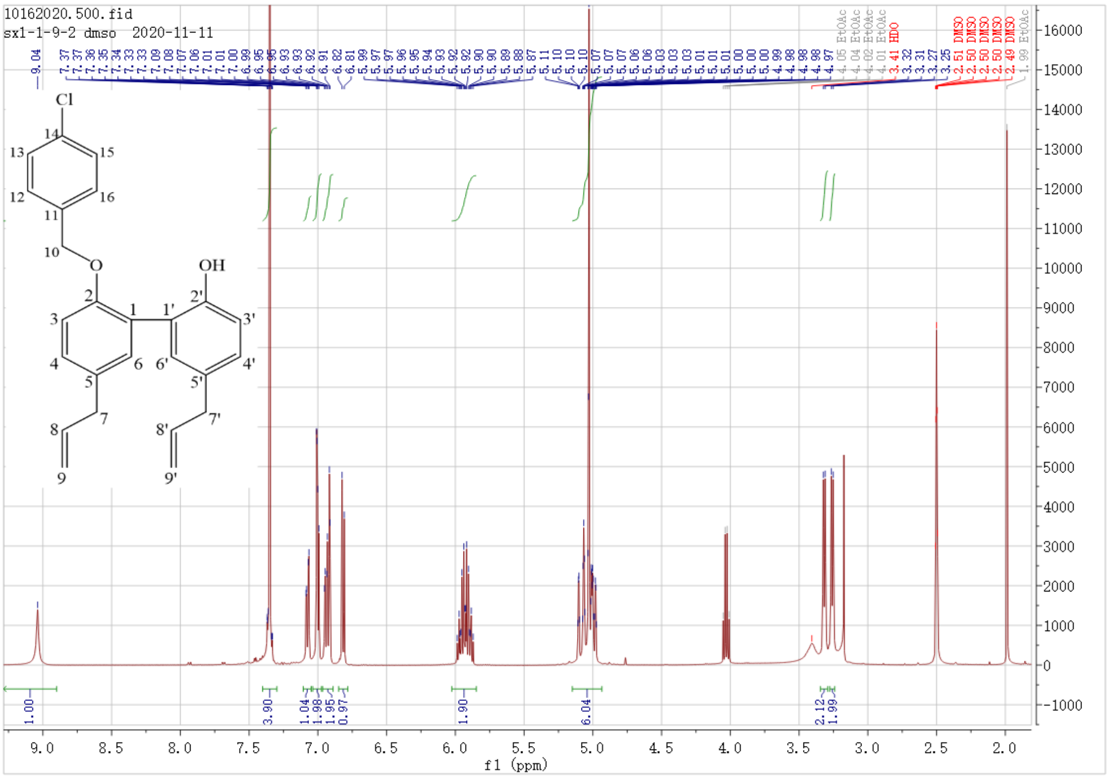

Figure S29 ^{1}H NMR spectrum of compound 9a

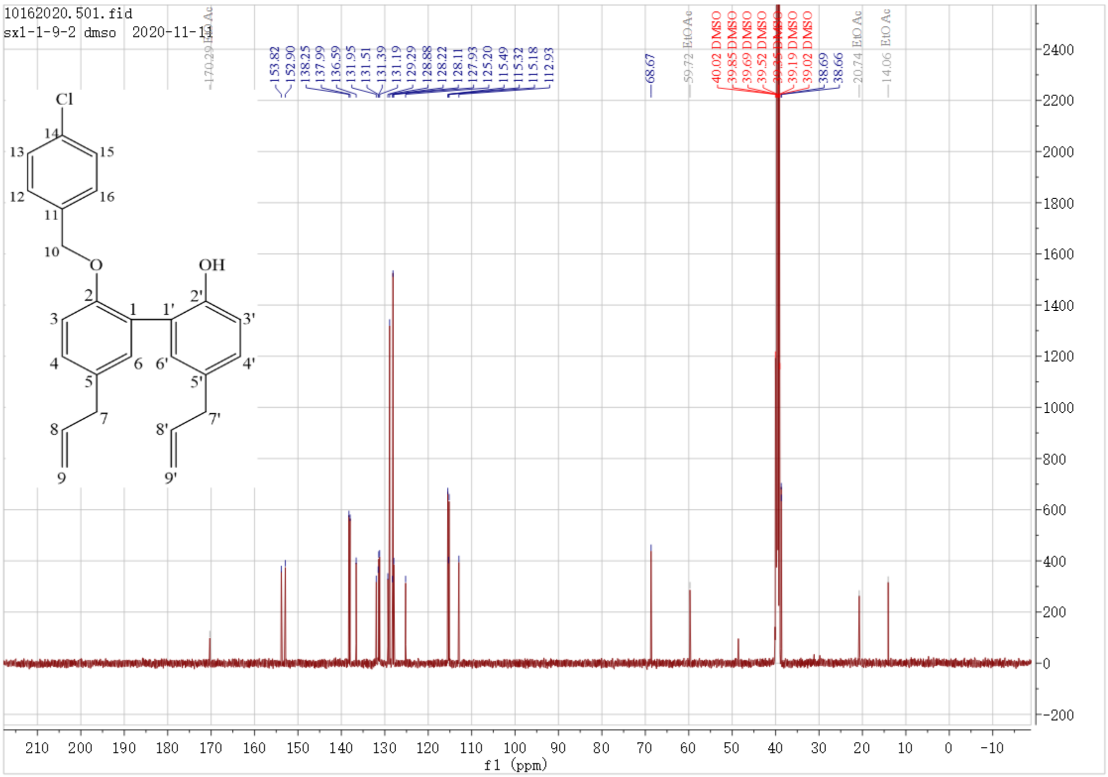

Figure S30 ^{13}C NMR spectrum of 9a

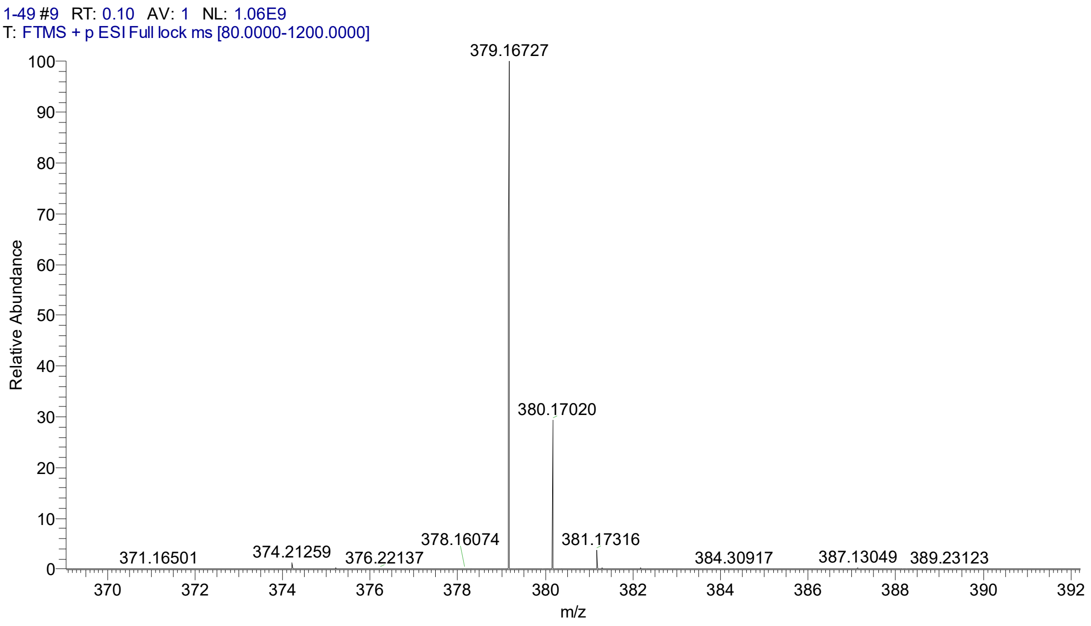

Figure S31 HR-ESI-MS spectrum of compound 3a

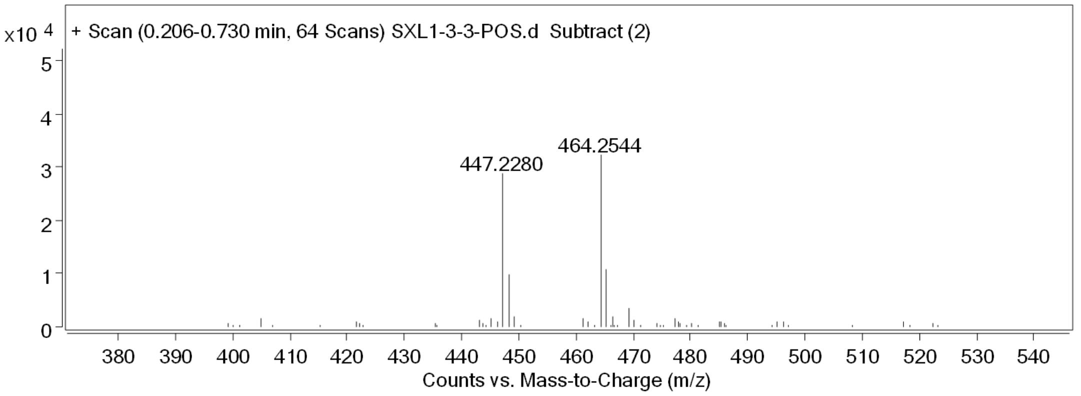

Figure S32 HR-ESI-MS spectrum of compound 3b

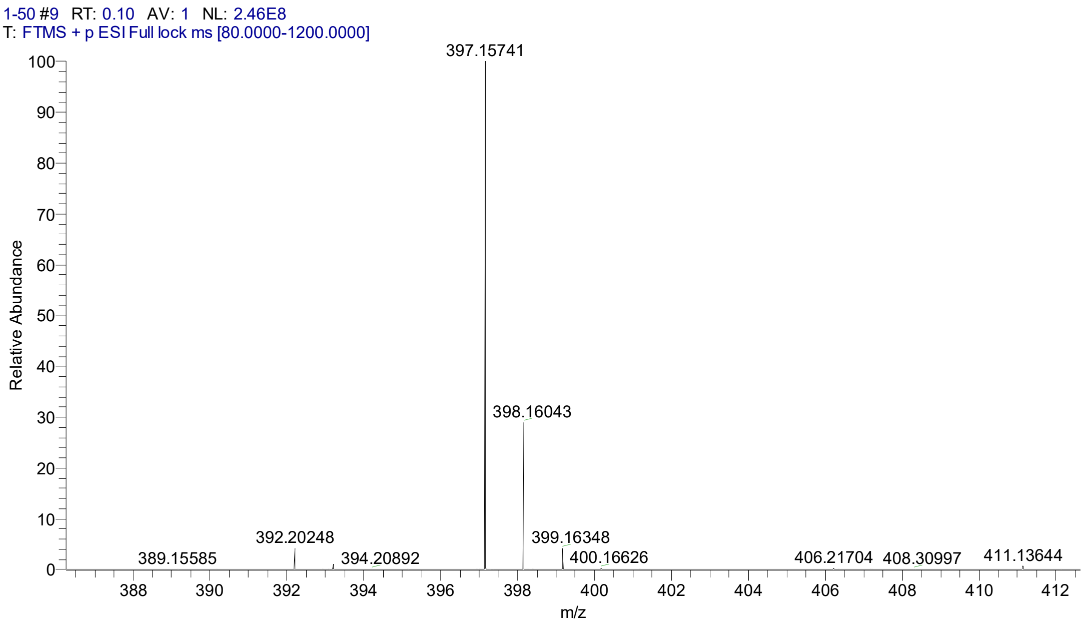

Figure S33 HR-ESI-MS spectrum of compound 4a

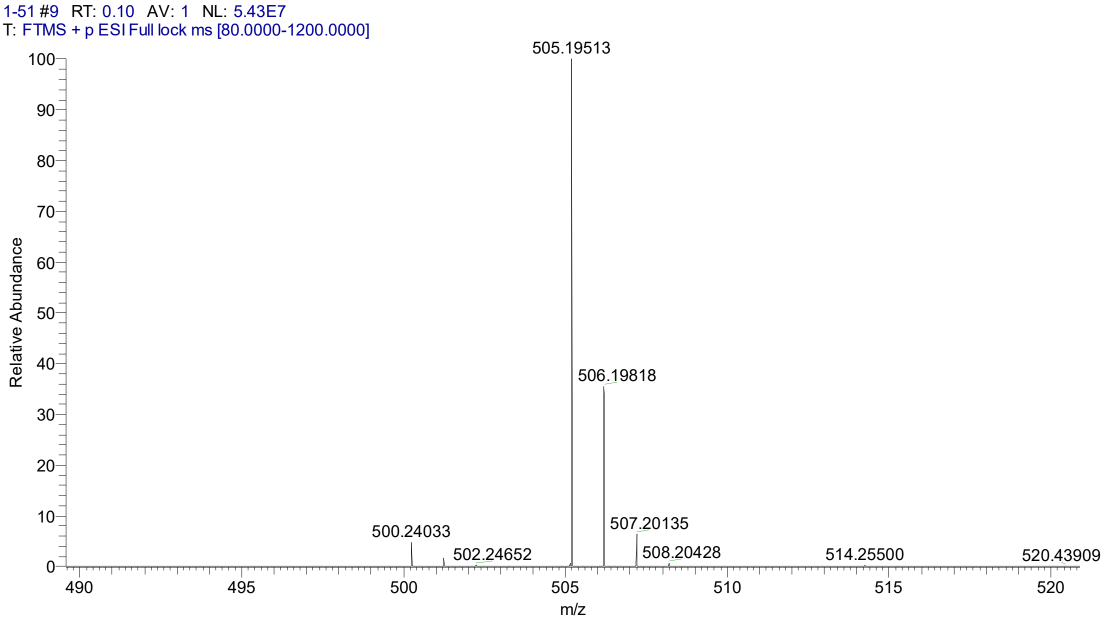

Figure S34 HR-ESI-MS spectrum of compound 4b

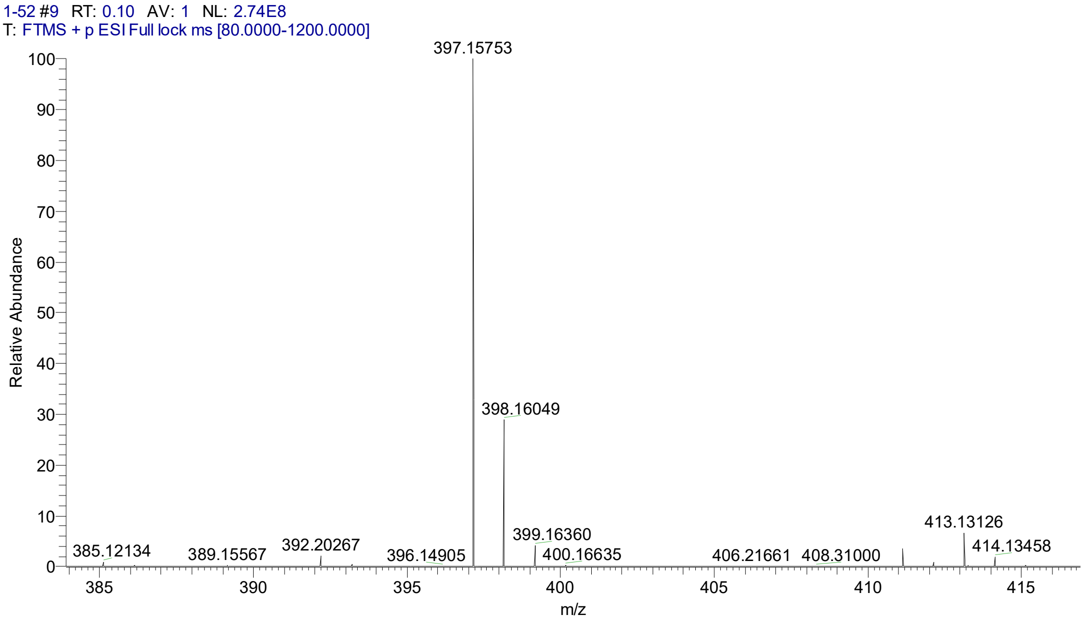

Figure S35 HR-ESI-MS spectrum of compound 5a

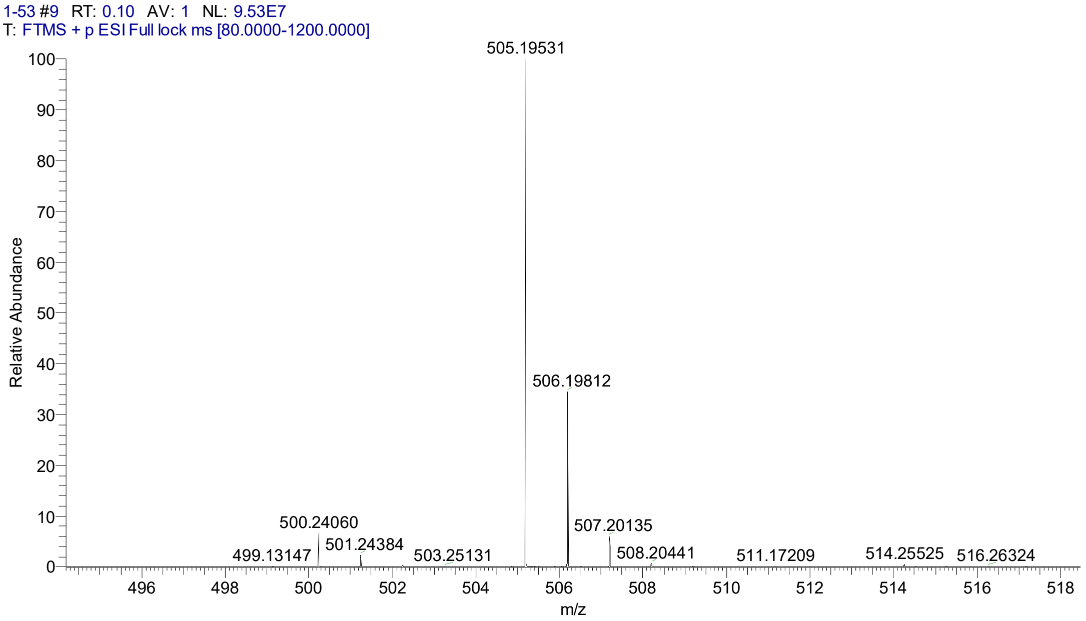

Figure S36 HR-ESI-MS spectrum of compound 5b

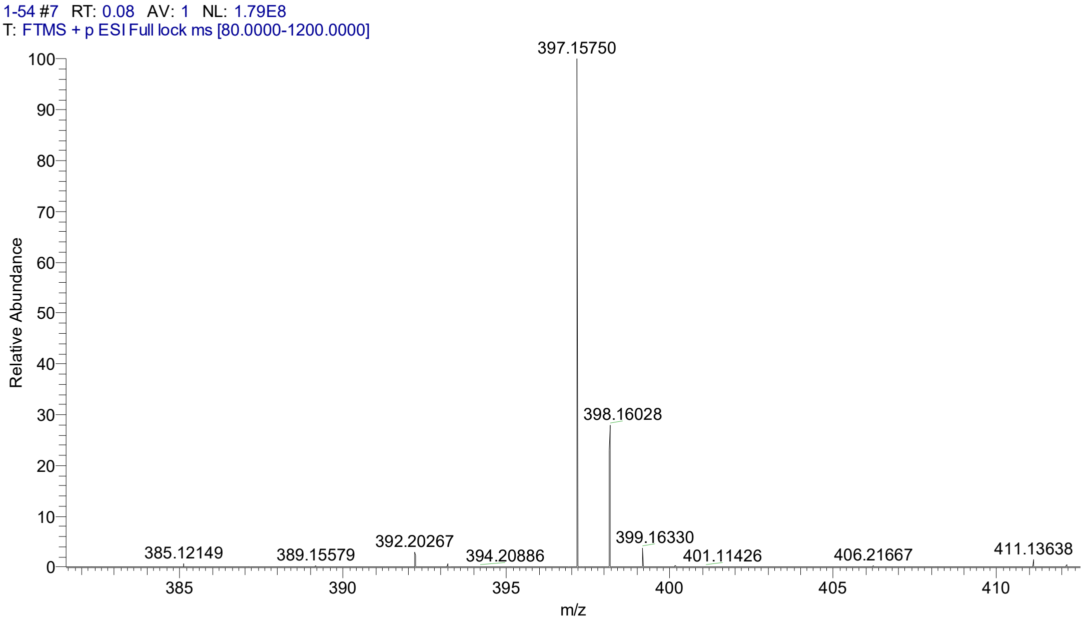

Figure S37 HR-ESI-MS spectrum of compound 6a

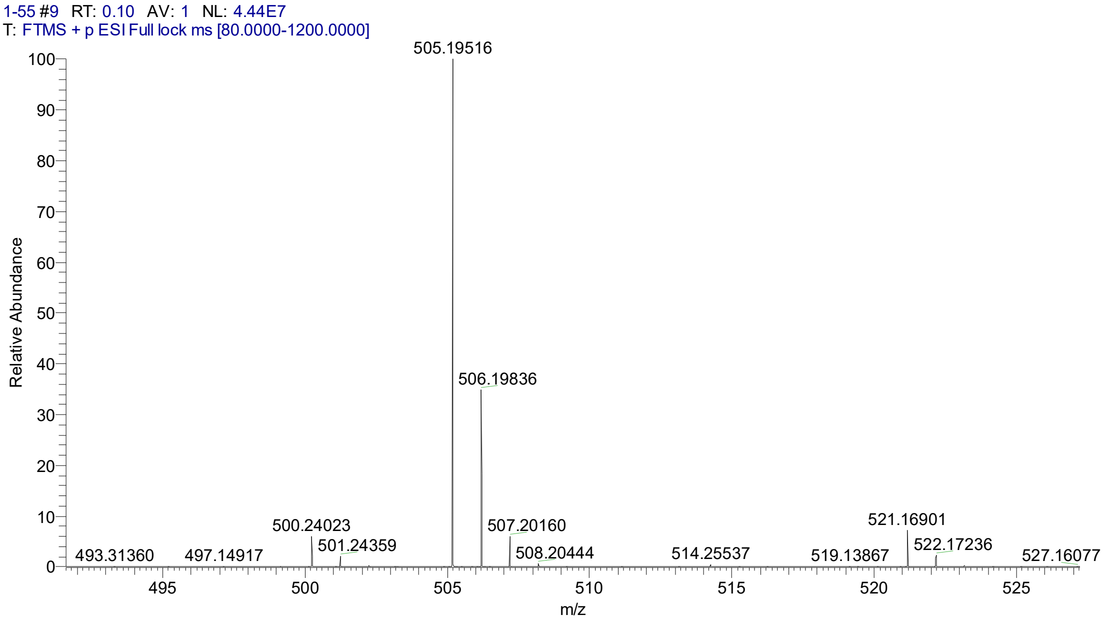

Figure S38 HR-ESI-MS spectrum of compound 6b

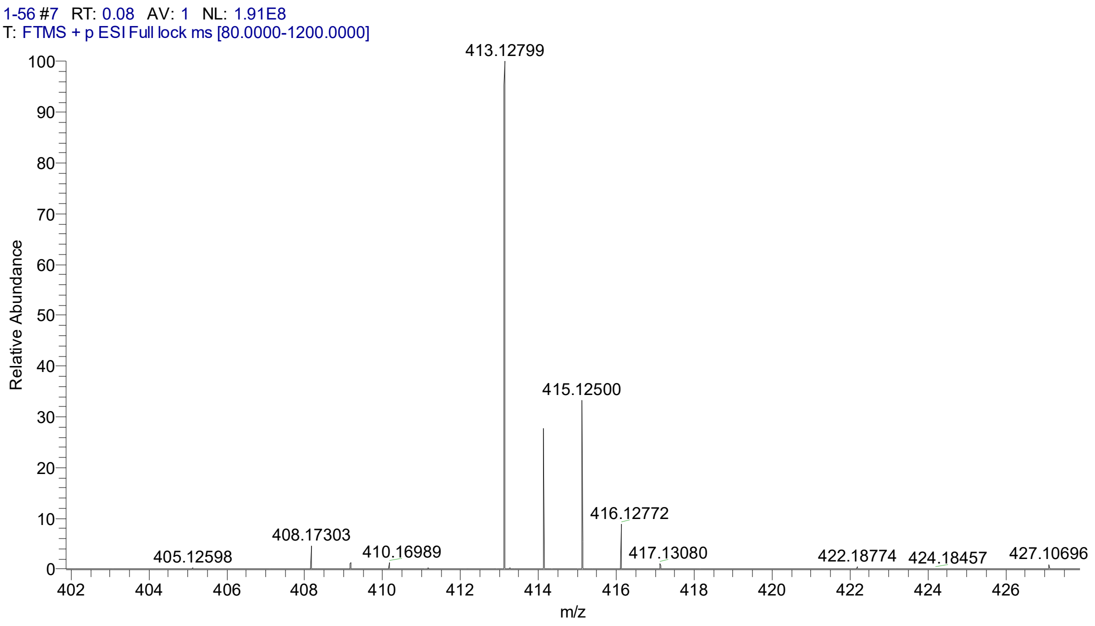

Figure S39 HR-ESI-MS spectrum of compound 7a

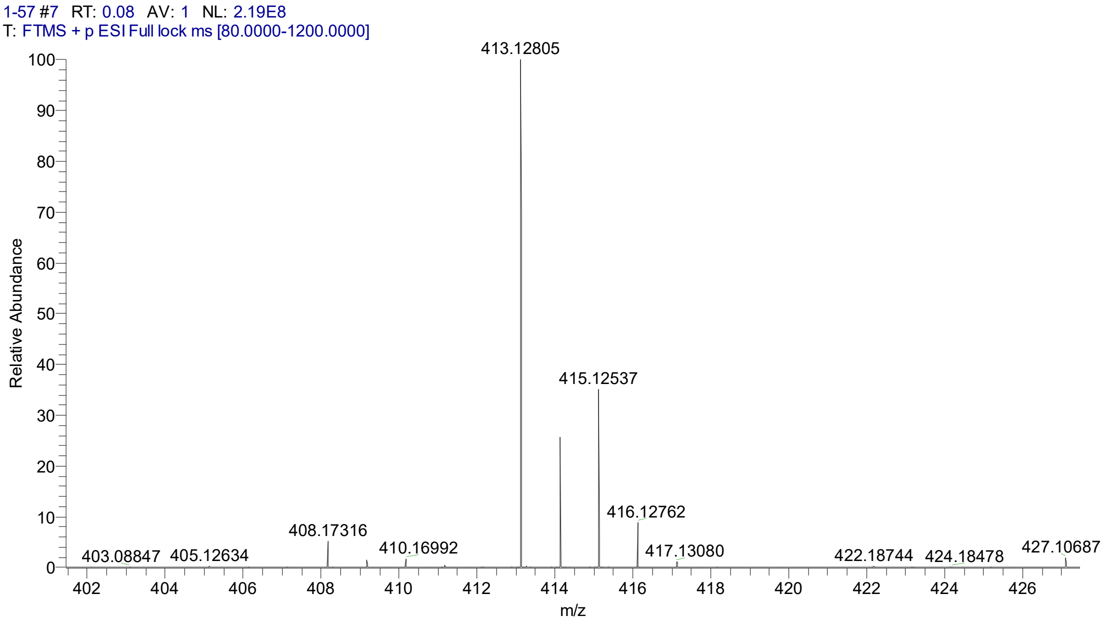

Figure S40 HR-ESI-MS spectrum of compound 8a

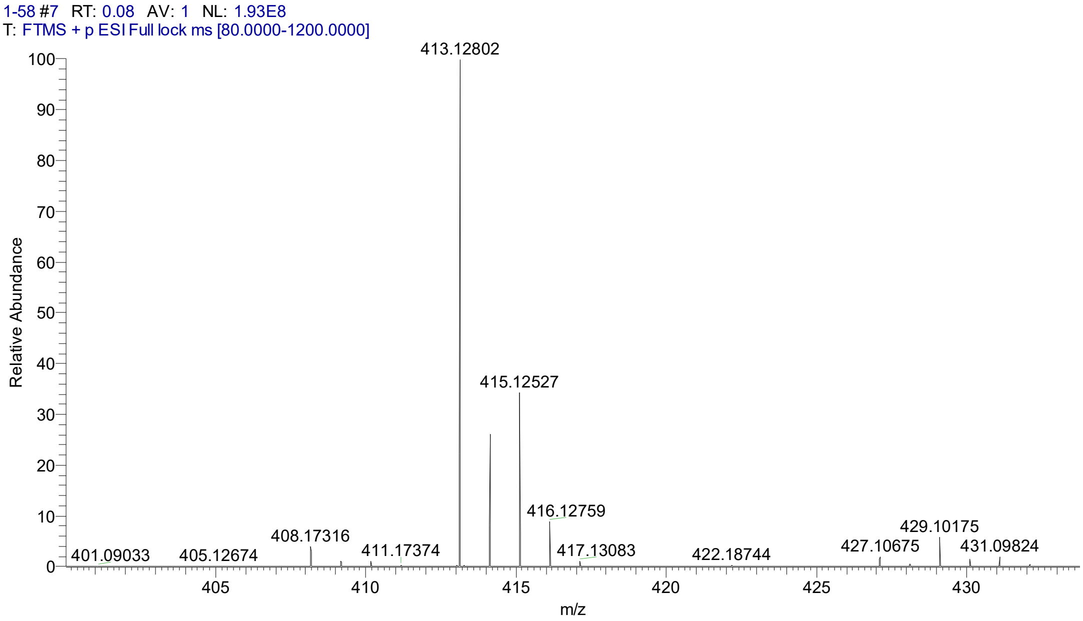

Figure S41 HR-ESI-MS spectrum of compound 9a
